# Photogenic Flies—A review of Chyb and Gompel's *Atlas of Drosophila Morphology: Wild-type and Classical Mutants*

**DOI:** 10.3389/fgene.2013.00147

**Published:** 2013-08-06

**Authors:** Andreas Prokop, Casey M. Bergman

**Affiliations:** Faculty of Life Sciences, University of ManchesterManchester, UK

For over a hundred years, visible mutations in genes of the fruitfly *Drosophila melanogaster* have been used to track chromosomal regions and gain insight into basic principles of biology (Morgan, 1910). These so-called “marker” mutations, many coming from the very early years of fly genetics, remain an omnipresent and irreplaceable part of the toolkit used in daily fly pushing activities in modern *Drosophila* laboratories. Generations of Drosophilists have learned about marker mutations from detailed descriptions and wonderfully instructive drawings in a series of printed catalogues, starting first with the “Dutch Book” (Morgan et al., 1925) and culminating with the famous Red Book (last updated in 1992) (Lindsley and Zimm, 1992). Much of this accumulated information is now accessible through FlyBase, however, all of these classic laboratory reference books are now out of print.

Chyb and Gompel's recently published *Atlas of Drosophila Morphology: Wild-type and Classical Mutants* (Academic Press) provides a timely and much-needed twenty-first century update to the classic line of mutant reference handbooks for *Drosophila* researchers. Instead of providing a comprehensive catalogue, the *Atlas* focuses on 70 of the most common marker mutations still in use today at an entirely new level of resolution made possible though the power of digital photography. The technical and aesthetic perfection of the images in the *Atlas* was achieved by using a custom-built fly photo studio equipped with light-emitting diodes, taking multiple images at different focal planes and assembling them together in imaging software. These images achieve a clarity and crispness that authentically reproduces the direct view of flies under the dissection microscope, far surpassing the utility (and, arguably, also the beauty) of previous black-and-white line drawings, and provide researchers with an entirely new dimension of illustration—color—that is so important for many eye and body markers, yet so difficult to describe in words. Collectively, the images in this book show that *Drosophila* are truly, in the authors' words, “photogenic flies.”

Like the famous “Learning to Fly” poster by Childress, Behringer and Halder (Wiley) that hangs in so many fly labs, and the recently issued photographs of marker mutants provided through FlyBase, the *Atlas* organizes marker mutations by anatomical structure, e.g. bristle, wing, other appendages, eye, and others. This organization by structure, which is further enhanced through summary photo plates, provides the didactic dimension of comparability that will be appreciated particularly by fly novices. Notably, the images are accompanied by extremely helpful information such as FlyBase entry, phenotypic variation, and similarities to other marker mutations. The *Atlas* is formatted so that easy-to-read textual information on the left page complements annotated images on the right, and it is spiral-bound so that it lays open flat unaided—all properties that enhance its practical value next to the microscope. The *Atlas* is also available in an e-book format for those more digitally inclined.

But the *Atlas* has far more to offer. It provides exquisite written and photographic descriptions of the wild-type morphology including bristles and appendages, as well as tips such as sexing of larvae or pupae, or the subtle parameters used for pupal staging. This section, however, could have benefitted from images of the larval mouth hook anatomy used to determine larval stages (Ashburner et al., 2005). A further very useful section describes the markers and chromosomal organization of the major balancer chromosomes, with simple pictograms that illustrate their cytology and photos of flies carrying combinations of markers for a selection of balancers. The closing section provides beautiful images of the adult morphology and distinguishing genitalia of the nine species in the *melanogaster* group. This echoes the inclusion of wild-type *D. melanogaster* strains in earlier catalogs (Bridges and Brehme, 1944; Lindsley and Grell, 1968) and highlights how this book feels as much like a field guide as it does like a laboratory handbook. These images of other *Drosophila* species are unfortunately not accompanied by the kind of textual descriptions that made the other parts of the book so accessible to novices and should be considered in future editions.

Like its predecessors, Chyb and Gompel's *Atlas* is a gift to the fly community that will undoubtedly become a standard addition to *Drosophila* labs around the world. As part of the modern cannon of *Drosophila* practical reference works (Greenspan, 2004; Ashburner et al., 2005; Markow and O'Grady, 2005; Roote and Prokop, 2013), the *Atlas* will serve an important role in helping new and experienced students of the fly to better understand the intricacies of this complex animal. But perhaps most importantly, this book provides a bridge between the classical and modern era of *Drosophila* research, allowing core knowledge about the fly to be transmitted more easily to new generations of researchers and help maintain *Drosophila* at the forefront of biological science into the future. The phrase “check in the *Atlas*!” will undoubtedly echo in the many *Drosophila* labs around the world for years to come.
